# The complete chloroplast genome of *Viburnum dilatatum* (Adoxaceae)

**DOI:** 10.1080/23802359.2021.1891988

**Published:** 2021-03-22

**Authors:** Xudong Wang, Xueqing Zhang, Daojie Li, Junshan Hou, Leijie Sheng, Guoyu Sun, Longfei Zhu, Zhuoqin Chen

**Affiliations:** a School of Architecture, North China University of Water Resources and Electric Power, Zhengzhou, PR China; b China Construction Seventh Engineering Division Corp. Ltd, Zhengzhou, PR China

**Keywords:** *Viburnum dilatatum*, chloroplast genome, Illumina sequencing, phylogenetic tree

## Abstract

*Viburnum dilatatum* Thunb. is a large deciduous tree of Adoxaceae. In this study, the chloroplast genome sequence of *V. dilatatum* is 158,392 bp, consisting of a large single-copy (LSC) region with 87,070 bp, a small single-copy (SSC) region with 18,242 bp , and two inverted repeat (IR) regions with 26,540 bp. The GC content in the chloroplast genome of *C. julianae* is 38.1%. The chloroplast genome of *V. dilatatum* contains 126 genes, including 83 protein-coding genes, 39 tRNA genes, and 4 rRNA genes. Phylogenetic tree showed that *V. dilatatum* was clustered with *V. utile*.

Viburnum is a genus of Adoxaceae, including more than 200 species, and widely distributed in temperate and subtropical regions of the world. It has been reported that the solvent extracts of *Viburnum dilatatum* Thunb. could be used as an anti-irritation ingredient (Kwon et al. 2010). And its fruit has the alpha-glucosidase inhibitory activities and the antihyperglycemic action (Kunihisal et al. 2006). It is closely related to *Viburnum erosum* Thunb. and *Viburnum wrightii* Miq., meanwhile its intermediate forms are often found, resulting in taxonomic confusions (Jongsun 2019). So our complete Chloroplast genome data of *V*. *dilatatum* can contribute to a better understanding of the evolution of Viburnum.

The fresh leaf samples of *V. dilatatum* were collected in Botanical Garden, Zhengzhou, China (N34449.1680; E1133211.7960). The voucher specimen was deposited at the Herbarium of Henan Agricultural University (voucher number:VD-20-0915). The total genomic DNA was extracted from fresh leaves of *V. dilatatum* using a modified CTAB method (Doyle and Doyle 1987). Sequencing was performed with the Illumina HiSeq2500 Platform (San Diego, CA). The raw reads were generated by Illumina paired-end sequencing after removing adapters. The low-quality sequences of raw reads used Fastp (https://github.com/OpenGene/Fastp) for quality control. Resultant clean reads were assembled by GetOrganelle pipeline version 1.6.3a (https://github.com/Kinggerm/GetOrganelle) with the gene from *Viburnum utile* (GenBank accession no. NC_032296) as the reference sequence. The genome was automatically annotated by using the CpGAVAS2 pipeline (http://www.herbalgenomics.org/cpgavas) (Shi et al. 2019) and start/stop codons and intron/exon boundaries were adjusted in Geneious 20.2.2 (https://www.geneious.com/).

The chloroplast genome sequences of *V. dilatatum* were submitted to NCBI, and the accession number was MW346666. The genome sequences of *V. dilatatum* is 158,392 bp, consisting of a large single-copy (LSC) region with 87,070 bp, a small single-copy (SSC) region with 18,242 bp, and two inverted repeat (IR) regions with 26,540 bp. The GC content in the chloroplast genome of *V. dilatatum* is 38.1%. The chloroplast genome of *V. dilatatum* contained 126 genes, including 83 protein-coding genes, 39 tRNA genes, and 4 rRNA genes.

The phylogenetic tree was constructed based on the genome sequences of *V. dilatatum* in RAxML version 8.2 (Stamatakis 2006) with 1000 bootstrap replicates. As shown in the phylogenetic tree (Figure 1), the sixteen Adoxaceae species were grouped into four clusters. One of the clusters was comprised of four genus *Viburnum* species, which *V. dilatatum* was closely related to *V. utile*. This result was similar to the previous phylogenetic trees based on chloroplast genome sequences of Adoxaceae (Wang et al. 2020; Zhao et al. 2020).

**Figure 1.**
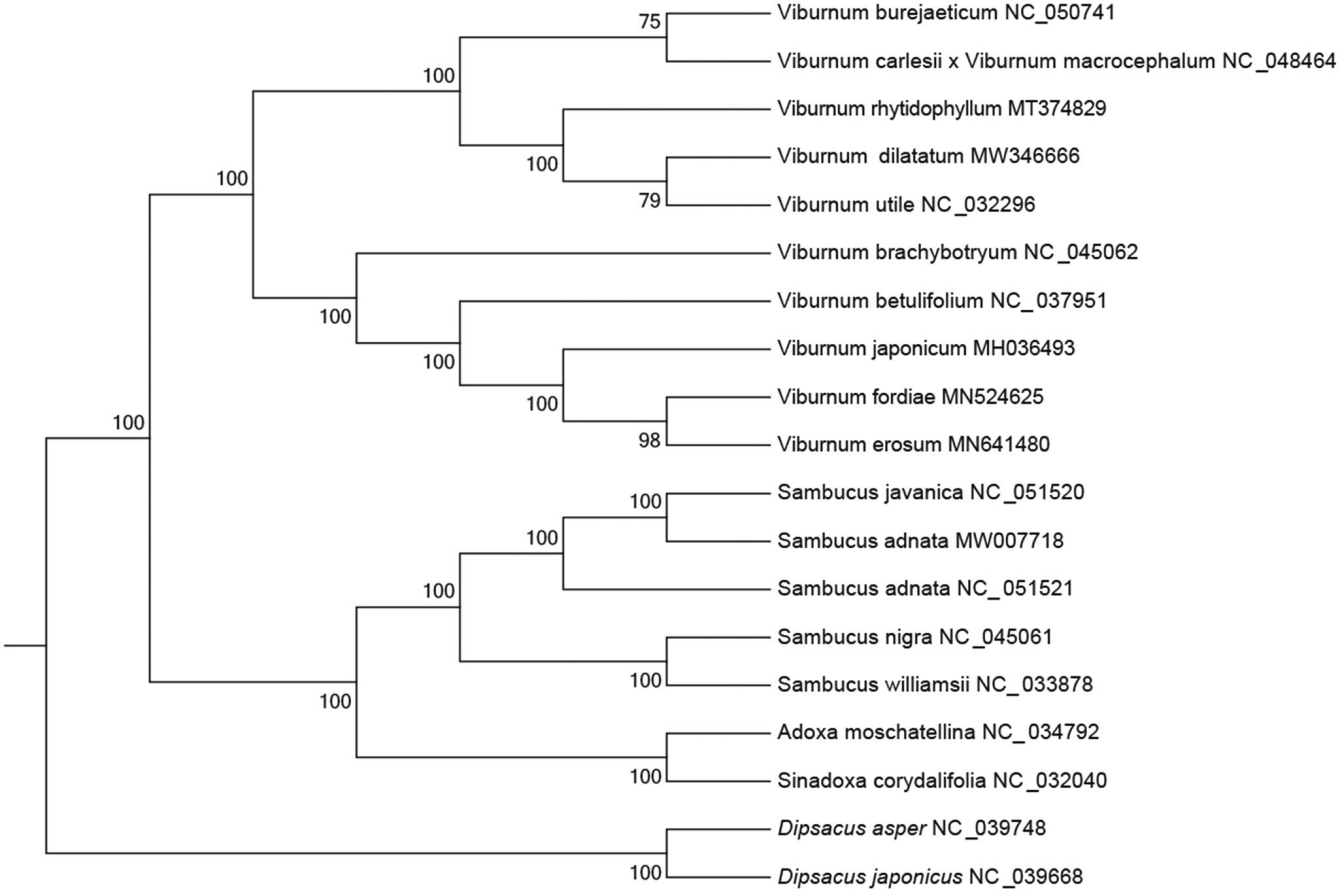
Maximum likelihood (ML) phylogenetic tree inferred from 16 plant chloroplast genomes. Numbers next to the branches are bootstrap support percentages.

## Data Availability

The genome sequence data that support the findings of this study are openly available in GenBank of NCBI at (https://www.ncbi.nlm.nih.gov/) under the accession no. MW346666. The associated BioProject, SRA, and Bio-Sample numbers are PRJNA670186, SRA: SRS7893946, and SAMN17100625, respectively.
